# Supplementary material for: The age of onset of substance use is related to the coping strategies to deal with treatment in men with substance use disorder
Source: PeerJ. 2017 Aug 15;5:e3660. doi: 10.7717/peerj.3660 (PMC5562142; doi:10.7717/peerj.3660)
Supplement: Data S1 [file peerj-05-3660-s001.pdf]

Groups	Age	Years_Educ	Marital_Sta	Economic_	Relatives_S	Relatives_F	Num_Suicid	One_subsa
1,00	51,00	8,00	3,00	3,00	1,00	0,00	0,00	1,00
1,00	25,00	10,00	3,00	3,00	0,00	0,00	0,00	1,00
1,00	23,00	12,00	0,00	3,00	0,00	0,00	0,00	1,00
1,00	24,00	12,00	0,00	4,00	0,00	0,00	1,00	0,00
1,00	28,00	10,00	2,00	4,00	0,00	0,00	0,00	0,00
1,00	33,00	6,00	1,00	5,00	0,00	0,00	0,00	0,00
1,00	26,00	12,00	0,00	3,00	0,00	0,00	0,00	0,00
1,00	52,00	12,00	2,00	1,00	0,00	1,00	0,00	1,00
1,00	31,00	8,00	0,00	3,00	0,00	1,00	0,00	0,00
1,00	36,00	6,00	3,00	3,00	0,00	0,00	0,00	0,00
1,00	28,00	10,00	0,00	3,00	1,00	0,00	0,00	0,00
1,00	31,00	9,00	0,00	3,00	0,00	0,00	2,00	0,00
1,00	33,00	10,00	0,00	5,00	0,00	0,00	0,00	0,00
1,00	20,00	8,00	0,00	4,00	0,00	0,00	0,00	1,00
1,00	37,00	6,00	3,00	5,00	0,00	0,00	0,00	0,00
1,00	55,00	12,00	3,00	1,00	0,00	1,00	1,00	1,00
1,00	29,00	10,00	3,00	1,00	0,00	0,00	0,00	0,00
1,00	54,00	10,00	2,00	3,00	0,00	0,00	0,00	0,00
1,00	40,00	8,00	2,00	2,00	0,00	0,00	0,00	0,00
1,00	39,00	8,00	2,00	4,00	0,00	0,00	0,00	0,00
1,00	22,00	12,00	0,00	3,00	0,00	0,00	0,00	0,00
1,00	28,00	18,00	0,00	5,00	0,00	0,00	0,00	0,00
1,00	23,00	12,00	1,00	4,00	1,00	1,00	0,00	1,00
1,00	32,00	8,00	0,00	2,00	1,00	0,00	0,00	0,00
1,00	30,00	15,00	3,00	5,00	0,00	0,00	0,00	1,00
1,00	33,00	18,00	0,00	5,00	0,00	0,00	0,00	0,00
1,00	31,00	12,00	1,00	4,00	1,00	0,00	0,00	0,00
1,00	28,00	12,00	1,00	2,00	0,00	0,00	0,00	0,00
1,00	37,00	12,00	3,00	2,00	0,00	0,00	0,00	1,00
1,00	53,00	12,00	2,00	2,00	0,00	0,00	0,00	0,00
1,00	36,00	15,00	1,00	1,00	0,00	0,00	0,00	0,00
1,00	28,00	8,00	2,00	2,00	0,00	0,00	0,00	0,00
1,00	43,00	12,00	3,00	1,00	0,00	0,00	1,00	0,00
1,00	29,00	8,00	0,00	4,00	1,00	0,00	0,00	1,00
1,00	30,00	10,00	0,00	4,00	0,00	0,00	0,00	0,00
1,00	27,00	10,00	1,00	4,00	0,00	0,00	1,00	0,00
1,00	43,00	12,00	3,00	3,00	0,00	1,00	3,00	0,00
1,00	36,00	10,00	3,00	1,00	0,00	1,00	0,00	0,00
1,00	39,00	10,00	1,00	1,00	1,00	1,00	0,00	0,00
1,00	27,00	12,00	0,00	2,00	1,00	0,00	0,00	0,00
1,00	30,00	10,00	0,00	2,00	1,00	1,00	0,00	0,00
1,00	34,00	12,00	0,00	3,00	0,00	1,00	2,00	0,00
1,00	28,00	6,00	0,00	1,00	0,00	0,00	0,00	0,00
1,00	32,00	10,00	0,00	3,00	0,00	0,00	0,00	0,00
1,00	35,00	6,00	0,00	3,00	0,00	0,00	0,00	0,00
1,00	30,00	12,00	0,00	2,00	0,00	0,00	0,00	0,00
1,00	29,00	10,00	0,00	3,00	1,00	1,00	0,00	0,00
1,00	33,00	8,00	0,00	1,00	1,00	0,00	2,00	0,00
1,00	41,00	12,00	0,00	3,00	1,00	0,00	0,00	0,00

1,00	23,00	10,00	0,00	3,00	1,00	1,00	0,00	0,00
1,00	36,00	10,00	0,00	4,00	1,00	1,00	0,00	0,00
1,00	38,00	10,00	0,00	4,00	0,00	0,00	0,00	1,00
1,00	26,00	8,00	0,00	3,00	0,00	0,00	0,00	0,00
1,00	30,00	10,00	3,00	4,00	0,00	0,00	0,00	0,00
1,00	53,00	10,00	1,00	1,00	0,00	0,00	0,00	0,00
1,00	25,00	8,00	0,00	1,00	0,00	0,00	0,00	0,00
1,00	39,00	12,00	2,00	4,00	0,00	0,00	0,00	0,00
1,00	28,00	10,00	0,00	4,00	0,00	0,00	0,00	0,00
1,00	37,00	10,00	3,00	2,00	1,00	1,00	1,00	0,00
1,00	39,00	6,00	0,00	1,00	0,00	1,00	4,00	0,00
2,00	38,00	10,00	1,00	4,00	0,00	0,00	0,00	0,00
2,00	49,00	12,00	3,00	3,00	0,00	0,00	0,00	1,00
2,00	55,00	16,00	2,00	5,00	0,00	0,00	0,00	1,00
2,00	43,00	8,00	1,00	3,00	0,00	0,00	1,00	0,00
2,00	32,00	12,00	2,00	3,00	0,00	0,00	1,00	0,00
2,00	33,00	10,00	0,00	4,00	0,00	0,00	0,00	1,00
2,00	38,00	12,00	0,00	4,00	0,00	0,00	3,00	0,00
2,00	55,00	16,00	3,00	5,00	0,00	1,00	#¡NULO!	0,00
2,00	35,00	12,00	3,00	5,00	0,00	1,00	0,00	0,00
2,00	36,00	12,00	0,00	3,00	0,00	1,00	0,00	1,00
2,00	30,00	12,00	1,00	5,00	1,00	0,00	0,00	0,00
2,00	42,00	8,00	0,00	5,00	0,00	0,00	0,00	1,00
2,00	36,00	10,00	0,00	3,00	1,00	0,00	0,00	1,00
2,00	54,00	8,00	3,00	1,00	1,00	1,00	0,00	0,00
2,00	40,00	10,00	0,00	5,00	0,00	0,00	0,00	1,00
2,00	39,00	8,00	0,00	2,00	0,00	0,00	0,00	0,00
2,00	38,00	6,00	3,00	3,00	0,00	1,00	0,00	0,00
2,00	34,00	6,00	1,00	3,00	0,00	0,00	0,00	0,00
2,00	29,00	12,00	0,00	4,00	0,00	0,00	0,00	0,00
2,00	43,00	6,00	2,00	2,00	0,00	0,00	0,00	0,00
2,00	26,00	8,00	0,00	3,00	0,00	1,00	0,00	0,00
2,00	29,00	12,00	0,00	4,00	0,00	0,00	0,00	1,00
2,00	53,00	10,00	1,00	1,00	0,00	0,00	0,00	0,00
2,00	33,00	10,00	0,00	1,00	0,00	0,00	0,00	0,00
2,00	39,00	8,00	2,00	2,00	0,00	0,00	0,00	0,00
2,00	51,00	8,00	0,00	4,00	0,00	1,00	1,00	1,00
2,00	32,00	12,00	0,00	3,00	0,00	0,00	0,00	0,00
2,00	46,00	12,00	2,00	2,00	0,00	1,00	1,00	0,00
2,00	29,00	12,00	0,00	2,00	0,00	0,00	0,00	1,00
2,00	41,00	8,00	3,00	1,00	0,00	0,00	0,00	0,00
2,00	26,00	12,00	0,00	2,00	0,00	0,00	0,00	1,00
2,00	27,00	12,00	0,00	2,00	1,00	0,00	0,00	0,00
2,00	29,00	12,00	0,00	2,00	0,00	0,00	0,00	1,00
2,00	42,00	10,00	2,00	2,00	0,00	1,00	0,00	0,00
2,00	52,00	10,00	3,00	2,00	0,00	0,00	0,00	0,00
2,00	30,00	10,00	0,00	2,00	0,00	0,00	0,00	0,00
2,00	46,00	12,00	3,00	2,00	0,00	0,00	0,00	1,00
2,00	34,00	6,00	0,00	2,00	0,00	1,00	0,00	0,00
2,00	38,00	10,00	0,00	2,00	0,00	0,00	0,00	0,00

2,00	40,00	12,00	3,00	2,00	0,00	0,00	0,00	0,00
2,00	36,00	12,00	3,00	2,00	0,00	0,00	0,00	0,00
2,00	32,00	12,00	0,00	2,00	0,00	0,00	0,00	1,00
2,00	40,00	8,00	0,00	4,00	0,00	1,00	1,00	0,00
2,00	40,00	8,00	2,00	2,00	0,00	0,00	0,00	1,00
2,00	42,00	10,00	3,00	3,00	0,00	1,00	0,00	0,00
2,00	41,00	10,00	0,00	1,00	1,00	0,00	1,00	0,00
2,00	37,00	10,00	2,00	2,00	1,00	1,00	0,00	0,00
2,00	38,00	10,00	0,00	3,00	0,00	1,00	0,00	1,00
2,00	39,00	12,00	2,00	3,00	0,00	0,00	1,00	0,00
2,00	31,00	12,00	0,00	3,00	0,00	1,00	0,00	0,00
2,00	38,00	10,00	3,00	1,00	0,00	0,00	0,00	0,00
2,00	29,00	12,00	0,00	3,00	0,00	0,00	0,00	0,00
2,00	39,00	12,00	1,00	1,00	0,00	1,00	0,00	0,00
2,00	35,00	6,00	0,00	4,00	0,00	1,00	0,00	0,00
2,00	55,00	14,00	3,00	1,00	0,00	0,00	0,00	1,00
2,00	47,00	15,00	3,00	3,00	0,00	0,00	0,00	1,00
2,00	35,00	8,00	0,00	3,00	0,00	0,00	0,00	0,00
2,00	36,00	16,00	2,00	5,00	0,00	0,00	0,00	0,00
2,00	35,00	14,00	0,00	4,00	0,00	0,00	0,00	0,00
2,00	29,00	10,00	0,00	4,00	0,00	0,00	0,00	0,00
2,00	37,00	10,00	0,00	3,00	0,00	0,00	0,00	0,00
2,00	39,00	6,00	0,00	3,00	0,00	1,00	0,00	0,00

Two_subst	Polydrug	Cocaine	Alcohol	Cannabis	Hallucinoge	Opioids	Sedatives	Age_OSU
0,00	0,00	0,00	1,00	0,00	0,00	0,00	0,00	16,00
0,00	0,00	0,00	0,00	1,00	1,00	0,00	0,00	16,00
0,00	0,00	0,00	0,00	1,00	0,00	0,00	0,00	16,00
0,00	1,00	0,00	1,00	1,00	1,00	0,00	0,00	14,00
1,00	0,00	0,00	1,00	1,00	0,00	0,00	0,00	12,00
1,00	0,00	1,00	1,00	0,00	0,00	0,00	0,00	16,00
1,00	0,00	1,00	1,00	1,00	0,00	0,00	0,00	16,00
0,00	0,00	0,00	1,00	0,00	0,00	0,00	0,00	14,00
1,00	0,00	1,00	1,00	0,00	0,00	0,00	0,00	16,00
1,00	0,00	1,00	1,00	0,00	0,00	0,00	0,00	15,00
0,00	1,00	1,00	1,00	1,00	0,00	1,00	0,00	16,00
0,00	1,00	1,00	1,00	1,00	1,00	0,00	0,00	15,00
0,00	1,00	1,00	1,00	1,00	0,00	0,00	0,00	16,00
0,00	0,00	0,00	0,00	0,00	1,00	0,00	0,00	15,00
1,00	0,00	1,00	0,00	1,00	0,00	0,00	0,00	14,00
0,00	0,00	0,00	1,00	0,00	0,00	0,00	0,00	16,00
1,00	0,00	1,00	1,00	0,00	0,00	0,00	0,00	15,00
0,00	1,00	1,00	1,00	1,00	0,00	0,00	0,00	16,00
0,00	1,00	1,00	1,00	1,00	1,00	0,00	0,00	16,00
1,00	0,00	1,00	1,00	0,00	0,00	0,00	0,00	16,00
0,00	1,00	1,00	1,00	1,00	0,00	0,00	0,00	14,00
1,00	0,00	1,00	1,00	0,00	0,00	0,00	0,00	15,00
0,00	0,00	0,00	0,00	1,00	0,00	0,00	0,00	16,00
0,00	1,00	1,00	0,00	1,00	1,00	0,00	0,00	16,00
0,00	0,00	0,00	0,00	1,00	0,00	0,00	0,00	15,00
0,00	1,00	1,00	1,00	1,00	0,00	0,00	0,00	15,00
0,00	1,00	1,00	1,00	1,00	0,00	0,00	0,00	16,00
1,00	0,00	1,00	0,00	0,00	0,00	0,00	0,00	16,00
0,00	0,00	1,00	0,00	0,00	0,00	0,00	0,00	12,00
1,00	0,00	1,00	1,00	0,00	0,00	0,00	0,00	16,00
0,00	1,00	1,00	1,00	1,00	0,00	0,00	0,00	16,00
0,00	1,00	1,00	1,00	1,00	0,00	0,00	0,00	14,00
0,00	1,00	1,00	1,00	0,00	0,00	1,00	0,00	16,00
0,00	0,00	1,00	0,00	0,00	0,00	0,00	0,00	14,00
1,00	0,00	1,00	1,00	0,00	0,00	0,00	0,00	14,00
0,00	1,00	1,00	0,00	1,00	1,00	0,00	0,00	14,00
1,00	0,00	1,00	1,00	0,00	0,00	0,00	0,00	14,00
0,00	1,00	1,00	1,00	1,00	0,00	0,00	0,00	16,00
0,00	1,00	1,00	1,00	0,00	0,00	1,00	0,00	15,00
0,00	1,00	1,00	1,00	1,00	1,00	0,00	0,00	16,00
0,00	1,00	1,00	1,00	1,00	0,00	0,00	0,00	16,00
0,00	1,00	1,00	1,00	0,00	0,00	1,00	0,00	16,00
0,00	1,00	1,00	1,00	1,00	1,00	0,00	0,00	14,00
0,00	1,00	1,00	1,00	1,00	0,00	0,00	0,00	14,00
0,00	1,00	1,00	1,00	1,00	0,00	1,00	1,00	14,00
0,00	1,00	1,00	1,00	1,00	1,00	0,00	0,00	13,00
0,00	1,00	1,00	1,00	1,00	0,00	0,00	0,00	15,00
0,00	1,00	1,00	0,00	1,00	0,00	1,00	1,00	13,00
0,00	1,00	1,00	1,00	1,00	1,00	1,00	1,00	15,00

1,00	0,00	0,00	1,00	1,00	0,00	0,00	0,00	14,00
1,00	0,00	1,00	1,00	0,00	0,00	0,00	0,00	17,00
0,00	0,00	1,00	0,00	0,00	0,00	0,00	0,00	16,00
0,00	1,00	1,00	1,00	1,00	0,00	0,00	0,00	14,00
1,00	0,00	1,00	1,00	0,00	0,00	0,00	0,00	14,00
1,00	0,00	1,00	1,00	0,00	0,00	0,00	0,00	16,00
0,00	1,00	1,00	1,00	1,00	0,00	0,00	0,00	16,00
1,00	0,00	1,00	0,00	0,00	0,00	1,00	0,00	16,00
0,00	1,00	1,00	1,00	1,00	1,00	1,00	0,00	14,00
0,00	1,00	1,00	1,00	1,00	1,00	0,00	0,00	14,00
0,00	1,00	1,00	1,00	1,00	1,00	1,00	0,00	12,00
0,00	1,00	1,00	1,00	0,00	0,00	1,00	0,00	17,00
0,00	0,00	1,00	0,00	0,00	0,00	0,00	0,00	18,00
0,00	0,00	0,00	1,00	0,00	0,00	0,00	0,00	18,00
1,00	0,00	1,00	1,00	0,00	0,00	0,00	0,00	19,00
0,00	1,00	1,00	0,00	1,00	1,00	0,00	0,00	18,00
0,00	0,00	0,00	1,00	0,00	0,00	0,00	0,00	18,00
1,00	0,00	1,00	1,00	0,00	0,00	0,00	0,00	26,00
0,00	1,00	1,00	1,00	1,00	0,00	0,00	1,00	40,00
1,00	0,00	1,00	1,00	0,00	0,00	0,00	0,00	30,00
0,00	0,00	1,00	0,00	0,00	0,00	0,00	0,00	20,00
1,00	0,00	1,00	1,00	0,00	0,00	0,00	0,00	23,00
0,00	0,00	1,00	0,00	0,00	0,00	0,00	0,00	32,00
0,00	0,00	1,00	0,00	0,00	0,00	0,00	0,00	27,00
0,00	1,00	1,00	1,00	1,00	0,00	0,00	0,00	40,00
0,00	0,00	1,00	0,00	0,00	0,00	0,00	0,00	19,00
1,00	0,00	1,00	1,00	0,00	0,00	0,00	0,00	19,00
0,00	1,00	1,00	1,00	1,00	1,00	0,00	0,00	17,00
1,00	0,00	1,00	1,00	0,00	0,00	0,00	0,00	23,00
1,00	0,00	1,00	1,00	0,00	0,00	0,00	0,00	19,00
1,00	0,00	1,00	1,00	0,00	0,00	0,00	0,00	17,00
1,00	0,00	1,00	1,00	0,00	0,00	0,00	0,00	21,00
0,00	0,00	1,00	0,00	0,00	0,00	0,00	0,00	21,00
0,00	1,00	1,00	1,00	1,00	0,00	0,00	0,00	20,00
0,00	1,00	1,00	1,00	1,00	0,00	0,00	0,00	18,00
1,00	0,00	1,00	1,00	0,00	0,00	0,00	0,00	19,00
0,00	0,00	0,00	1,00	0,00	0,00	0,00	0,00	19,00
1,00	0,00	1,00	1,00	0,00	0,00	0,00	0,00	23,00
1,00	0,00	1,00	0,00	1,00	0,00	0,00	0,00	21,00
0,00	0,00	1,00	0,00	0,00	0,00	0,00	0,00	26,00
0,00	1,00	1,00	1,00	1,00	0,00	1,00	0,00	20,00
0,00	0,00	1,00	0,00	0,00	0,00	0,00	0,00	24,00
0,00	1,00	1,00	1,00	1,00	0,00	0,00	0,00	22,00
0,00	0,00	1,00	0,00	0,00	0,00	0,00	0,00	27,00
1,00	0,00	1,00	1,00	0,00	0,00	0,00	0,00	20,00
1,00	0,00	1,00	1,00	0,00	0,00	0,00	0,00	29,00
1,00	0,00	1,00	1,00	0,00	0,00	0,00	0,00	21,00
0,00	0,00	1,00	0,00	0,00	0,00	0,00	0,00	42,00
0,00	1,00	1,00	1,00	0,00	0,00	0,00	0,00	30,00
1,00	0,00	1,00	1,00	0,00	0,00	0,00	0,00	20,00

1,00	0,00	1,00	1,00	0,00	0,00	0,00	0,00	30,00
1,00	0,00	1,00	1,00	0,00	0,00	0,00	0,00	34,00
0,00	0,00	1,00	0,00	0,00	0,00	0,00	0,00	27,00
0,00	1,00	1,00	1,00	1,00	0,00	1,00	0,00	19,00
0,00	0,00	1,00	0,00	0,00	0,00	0,00	0,00	19,00
0,00	1,00	1,00	1,00	0,00	0,00	1,00	0,00	23,00
0,00	1,00	1,00	1,00	1,00	1,00	0,00	0,00	16,00
0,00	1,00	1,00	1,00	1,00	1,00	1,00	0,00	18,00
0,00	0,00	0,00	0,00	0,00	0,00	1,00	0,00	25,00
1,00	0,00	1,00	1,00	0,00	0,00	0,00	0,00	18,00
0,00	1,00	1,00	0,00	1,00	1,00	0,00	0,00	17,00
1,00	0,00	1,00	1,00	0,00	0,00	0,00	0,00	20,00
1,00	0,00	1,00	1,00	0,00	0,00	0,00	0,00	22,00
0,00	1,00	1,00	0,00	0,00	0,00	0,00	0,00	17,00
1,00	0,00	1,00	0,00	0,00	0,00	1,00	0,00	20,00
0,00	0,00	1,00	0,00	0,00	0,00	0,00	0,00	45,00
0,00	0,00	1,00	0,00	0,00	0,00	0,00	0,00	40,00
0,00	1,00	1,00	1,00	1,00	0,00	0,00	0,00	30,00
0,00	1,00	1,00	1,00	1,00	0,00	0,00	0,00	18,00
1,00	0,00	1,00	1,00	0,00	0,00	0,00	0,00	22,00
0,00	1,00	1,00	1,00	1,00	0,00	0,00	1,00	20,00
1,00	0,00	1,00	1,00	0,00	0,00	0,00	0,00	19,00
0,00	1,00	1,00	1,00	1,00	1,00	1,00	0,00	17,00

Years_Dura	Tipology_T	Medicatior	Months_Al	Past_Treat	Num_Relap	None_Rela	One_Relap	Two_Relap
33,00	2,00	0,00	24,00	#¡NULO!	1,00	0,00	1,00	0,00
8,17	2,00	0,00	10,00	#¡NULO!	1,00	0,00	1,00	0,00
6,17	2,00	1,00	10,00	#¡NULO!	1,00	0,00	1,00	0,00
9,75	2,00	0,00	3,00	#¡NULO!	3,00	0,00	0,00	0,00
15,67	2,00	0,00	4,00	#¡NULO!	0,00	1,00	0,00	0,00
16,33	2,00	0,00	8,00	#¡NULO!	3,00	0,00	0,00	0,00
9,50	2,00	1,00	6,00	1,00	0,00	1,00	0,00	0,00
37,08	2,00	0,00	11,00	1,00	1,00	0,00	1,00	0,00
14,33	2,00	0,00	8,00	0,00	0,00	1,00	0,00	0,00
20,17	2,00	0,00	10,00	1,00	0,00	1,00	0,00	0,00
11,50	2,00	4,00	6,00	1,00	0,00	1,00	0,00	0,00
15,50	2,00	1,00	6,00	1,00	0,00	1,00	0,00	0,00
16,50	2,00	2,00	6,00	1,00	2,00	0,00	0,00	1,00
4,67	2,00	0,00	4,00	0,00	0,00	1,00	0,00	0,00
22,67	2,00	0,00	4,00	0,00	2,00	0,00	0,00	1,00
38,75	2,00	0,00	3,00	0,00	3,00	0,00	0,00	0,00
13,67	1,00	0,00	4,00	1,00	0,00	1,00	0,00	0,00
37,33	1,00	1,00	8,00	0,00	3,00	0,00	0,00	0,00
23,17	1,00	1,00	10,00	1,00	3,00	0,00	0,00	0,00
22,17	1,00	2,00	10,00	0,00	0,00	1,00	0,00	0,00
7,50	1,00	0,00	6,00	0,00	2,00	0,00	0,00	1,00
12,50	1,00	0,00	6,00	1,00	0,00	1,00	0,00	0,00
6,70	1,00	2,00	4,00	1,00	0,00	1,00	0,00	0,00
15,50	1,00	2,00	6,00	0,00	0,00	1,00	0,00	0,00
14,17	1,00	0,00	10,00	0,00	0,00	1,00	0,00	0,00
17,50	1,00	0,00	6,00	0,00	2,00	0,00	0,00	1,00
14,50	1,00	2,00	6,00	1,00	0,00	1,00	0,00	0,00
11,08	1,00	0,00	11,00	0,00	0,00	1,00	0,00	0,00
24,58	1,00	0,00	5,00	0,00	0,00	1,00	0,00	0,00
35,50	1,00	0,00	18,00	0,00	0,00	1,00	0,00	0,00
19,33	1,00	1,00	8,00	1,00	1,00	0,00	1,00	0,00
12,83	1,00	0,00	14,00	0,00	0,00	1,00	0,00	0,00
26,67	2,00	3,00	4,00	0,00	0,00	1,00	0,00	0,00
14,58	2,00	4,00	5,00	1,00	0,00	1,00	0,00	0,00
15,67	2,00	0,00	4,00	0,00	0,00	1,00	0,00	0,00
12,00	2,00	2,00	12,00	1,00	0,00	1,00	0,00	0,00
28,00	2,00	1,00	12,00	1,00	3,00	0,00	0,00	0,00
19,08	2,00	0,00	11,00	0,00	0,00	1,00	0,00	0,00
23,67	2,00	1,00	4,00	1,00	2,00	0,00	0,00	1,00
9,67	2,00	0,00	16,00	1,00	2,00	0,00	0,00	1,00
13,00	2,00	0,00	12,00	0,00	2,00	0,00	0,00	1,00
17,67	2,00	0,00	4,00	0,00	0,00	1,00	0,00	0,00
13,00	2,00	0,00	12,00	0,00	2,00	0,00	0,00	1,00
17,58	2,00	0,00	5,00	1,00	0,00	1,00	0,00	0,00
20,58	2,00	1,00	5,00	1,00	0,00	1,00	0,00	0,00
16,58	2,00	0,00	5,00	0,00	0,00	1,00	0,00	0,00
13,50	2,00	0,00	6,00	1,00	3,00	0,00	0,00	0,00
19,42	2,00	3,00	7,00	0,00	1,00	0,00	1,00	0,00
25,67	2,00	0,00	4,00	1,00	0,00	1,00	0,00	0,00

8,67	2,00	0,00	4,00	1,00	0,00	1,00	0,00	0,00
18,67	2,00	0,00	4,00	0,00	2,00	0,00	0,00	1,00
21,67	2,00	0,00	4,00	0,00	0,00	1,00	0,00	0,00
11,58	2,00	0,00	5,00	1,00	0,00	1,00	0,00	0,00
15,67	2,00	0,00	4,00	1,00	3,00	0,00	0,00	0,00
36,58	2,00	0,00	5,00	1,00	0,00	1,00	0,00	0,00
8,17	2,00	0,00	10,00	1,00	0,00	1,00	0,00	0,00
21,00	2,00	0,00	24,00	0,00	0,00	1,00	0,00	0,00
13,42	2,00	0,00	7,00	1,00	1,00	0,00	1,00	0,00
22,83	2,00	3,00	2,00	0,00	3,00	0,00	0,00	0,00
26,67	2,00	1,00	4,00	1,00	1,00	0,00	1,00	0,00
19,00	2,00	0,00	24,00	#¡NULO!	3,00	0,00	0,00	0,00
30,67	1,00	0,00	4,00	#¡NULO!	3,00	0,00	0,00	0,00
36,33	1,00	1,00	8,00	#¡NULO!	3,00	0,00	0,00	0,00
22,33	1,00	0,00	20,00	#¡NULO!	0,00	1,00	0,00	0,00
12,50	1,00	0,00	18,00	#¡NULO!	3,00	0,00	0,00	0,00
14,75	2,00	0,00	3,00	#¡NULO!	0,00	1,00	0,00	0,00
11,33	2,00	0,00	8,00	#¡NULO!	2,00	0,00	0,00	1,00
14,58	1,00	0,00	5,00	#¡NULO!	3,00	0,00	0,00	0,00
4,08	1,00	0,00	11,00	#¡NULO!	1,00	0,00	1,00	0,00
15,42	1,00	0,00	7,00	#¡NULO!	0,00	1,00	0,00	0,00
6,67	2,00	0,00	4,00	#¡NULO!	0,00	1,00	0,00	0,00
9,67	2,00	0,00	4,00	#¡NULO!	1,00	0,00	1,00	0,00
8,67	2,00	1,00	4,00	#¡NULO!	1,00	0,00	1,00	0,00
13,50	1,00	0,00	6,00	#¡NULO!	1,00	0,00	1,00	0,00
20,67	2,00	0,00	4,00	#¡NULO!	3,00	0,00	0,00	0,00
19,10	1,00	0,00	11,00	0,00	0,00	1,00	0,00	0,00
18,00	1,00	1,00	36,00	1,00	2,00	0,00	0,00	1,00
10,42	2,00	2,00	7,00	1,00	0,00	1,00	0,00	0,00
9,25	2,00	1,00	9,00	1,00	0,00	1,00	0,00	0,00
25,00	2,00	1,00	12,00	1,00	0,00	1,00	0,00	0,00
4,58	2,00	1,00	5,00	1,00	1,00	0,00	1,00	0,00
7,67	2,00	0,00	4,00	1,00	0,00	1,00	0,00	0,00
32,50	1,00	1,00	6,00	1,00	2,00	0,00	0,00	1,00
14,58	1,00	0,00	5,00	1,00	2,00	0,00	0,00	1,00
19,33	1,00	0,00	8,00	0,00	3,00	0,00	0,00	0,00
31,42	1,00	1,00	7,00	1,00	3,00	0,00	0,00	0,00
8,50	1,00	#¡NULO!	6,00	1,00	0,00	1,00	0,00	0,00
24,75	1,00	0,00	3,00	0,00	0,00	1,00	0,00	0,00
2,42	1,00	0,00	7,00	0,00	0,00	1,00	0,00	0,00
20,50	1,00	0,00	6,00	0,00	0,00	1,00	0,00	0,00
1,33	1,00	0,00	8,00	0,00	0,00	1,00	0,00	0,00
4,33	1,00	0,00	8,00	0,00	1,00	0,00	1,00	0,00
1,42	1,00	0,00	7,00	0,00	0,00	1,00	0,00	0,00
21,08	1,00	1,00	11,00	0,00	0,00	1,00	0,00	0,00
21,50	1,00	0,00	18,00	1,00	0,00	1,00	0,00	0,00
8,33	1,00	0,00	8,00	1,00	1,00	0,00	1,00	0,00
2,58	1,00	0,00	17,00	0,00	0,00	1,00	0,00	0,00
2,92	1,00	0,00	13,00	0,00	0,00	1,00	0,00	0,00
17,08	1,00	0,00	11,00	0,00	0,00	1,00	0,00	0,00

8,67	1,00	0,00	16,00	0,00	0,00	1,00	0,00	0,00
1,42	1,00	0,00	7,00	0,00	0,00	1,00	0,00	0,00
4,08	1,00	0,00	11,00	0,00	0,00	1,00	0,00	0,00
20,50	2,00	0,00	6,00	1,00	3,00	0,00	0,00	0,00
20,58	2,00	0,00	5,00	1,00	1,00	0,00	1,00	0,00
18,58	2,00	2,00	5,00	1,00	0,00	1,00	0,00	0,00
24,50	2,00	2,00	6,00	0,00	1,00	0,00	1,00	0,00
18,00	2,00	2,00	12,00	1,00	1,00	0,00	1,00	0,00
11,67	2,00	0,00	16,00	0,00	3,00	0,00	0,00	0,00
20,33	2,00	1,00	8,00	1,00	1,00	0,00	1,00	0,00
13,33	2,00	0,00	8,00	1,00	0,00	1,00	0,00	0,00
17,67	2,00	0,00	4,00	1,00	1,00	0,00	1,00	0,00
6,67	2,00	0,00	4,00	1,00	0,00	1,00	0,00	0,00
21,67	2,00	1,00	4,00	0,00	0,00	1,00	0,00	0,00
14,67	2,00	2,00	4,00	0,00	0,00	1,00	0,00	0,00
9,58	2,00	0,00	5,00	0,00	0,00	1,00	0,00	0,00
6,67	2,00	0,00	4,00	0,00	1,00	0,00	1,00	0,00
4,25	2,00	0,00	9,00	0,00	0,00	1,00	0,00	0,00
17,50	2,00	0,00	6,00	1,00	0,00	1,00	0,00	0,00
12,67	2,00	0,00	7,00	1,00	0,00	1,00	0,00	0,00
8,25	2,00	1,00	9,00	1,00	0,00	1,00	0,00	0,00
17,33	2,00	0,00	8,00	0,00	0,00	1,00	0,00	0,00
21,17	2,00	0,00	10,00	0,00	0,00	1,00	0,00	0,00

Polyrelapse	ICG	CSI1_PS	CSI1_CR	CSI1_SS	CSI1_EE	CSI1_PA	CSI1_WT	CSI1_SW
0,00	1,00	20,00	12,00	17,00	20,00	8,00	16,00	3,00
0,00	1,00	14,00	7,00	13,00	11,00	0,00	17,00	2,00
0,00	4,00	11,00	9,00	10,00	9,00	17,00	18,00	15,00
1,00	2,00	19,00	13,00	12,00	15,00	1,00	17,00	3,00
0,00	3,00	14,00	9,00	0,00	5,00	4,00	20,00	7,00
1,00	3,00	6,00	15,00	10,00	13,00	7,00	19,00	1,00
0,00	3,00	7,00	14,00	14,00	12,00	9,00	12,00	6,00
0,00	3,00	14,00	8,00	5,00	8,00	7,00	17,00	10,00
0,00	2,00	15,00	6,00	4,00	9,00	16,00	9,00	14,00
0,00	3,00	20,00	4,00	12,00	20,00	0,00	20,00	8,00
0,00	3,00	15,00	8,00	5,00	16,00	5,00	18,00	4,00
0,00	6,00	14,00	14,00	13,00	8,00	17,00	15,00	15,00
0,00	2,00	8,00	14,00	10,00	18,00	14,00	17,00	13,00
0,00	2,00	19,00	12,00	14,00	8,00	4,00	15,00	7,00
0,00	3,00	15,00	7,00	6,00	14,00	0,00	6,00	13,00
1,00	2,00	18,00	3,00	11,00	16,00	4,00	9,00	9,00
0,00	1,00	14,00	12,00	12,00	6,00	6,00	12,00	6,00
1,00	2,00	19,00	15,00	17,00	4,00	2,00	11,00	7,00
1,00	1,00	20,00	17,00	16,00	17,00	9,00	20,00	8,00
0,00	4,00	10,00	2,00	4,00	8,00	3,00	17,00	17,00
0,00	1,00	11,00	4,00	8,00	3,00	6,00	7,00	17,00
0,00	3,00	8,00	6,00	9,00	12,00	6,00	15,00	14,00
0,00	4,00	6,00	8,00	13,00	9,00	5,00	19,00	9,00
0,00	3,00	18,00	15,00	18,00	16,00	6,00	19,00	16,00
0,00	1,00	6,00	2,00	2,00	5,00	0,00	16,00	18,00
0,00	3,00	15,00	6,00	4,00	9,00	16,00	9,00	14,00
0,00	2,00	10,00	6,00	7,00	2,00	10,00	15,00	16,00
0,00	3,00	17,00	10,00	12,00	7,00	12,00	2,00	1,00
0,00	#¡NULO!	17,00	13,00	10,00	11,00	7,00	16,00	10,00
0,00	2,00	9,00	12,00	14,00	13,00	17,00	11,00	11,00
0,00	1,00	7,00	0,00	0,00	12,00	3,00	2,00	11,00
0,00	4,00	7,00	9,00	17,00	7,00	8,00	16,00	4,00
0,00	2,00	16,00	20,00	20,00	20,00	1,00	20,00	4,00
0,00	5,00	15,00	5,00	16,00	12,00	1,00	16,00	10,00
0,00	3,00	6,00	8,00	20,00	9,00	5,00	18,00	10,00
0,00	4,00	17,00	5,00	10,00	12,00	2,00	17,00	8,00
1,00	5,00	10,00	7,00	11,00	17,00	4,00	20,00	6,00
0,00	2,00	16,00	16,00	14,00	16,00	14,00	19,00	20,00
0,00	1,00	11,00	5,00	14,00	11,00	6,00	10,00	6,00
0,00	3,00	17,00	20,00	16,00	20,00	0,00	20,00	16,00
0,00	4,00	2,00	5,00	2,00	9,00	11,00	20,00	16,00
0,00	4,00	15,00	9,00	14,00	3,00	1,00	18,00	8,00
0,00	4,00	15,00	19,00	4,00	9,00	15,00	16,00	16,00
0,00	3,00	14,00	13,00	8,00	9,00	1,00	8,00	3,00
0,00	4,00	11,00	4,00	6,00	3,00	6,00	10,00	8,00
0,00	2,00	8,00	7,00	9,00	3,00	15,00	13,00	11,00
1,00	1,00	10,00	4,00	17,00	10,00	12,00	9,00	11,00
0,00	5,00	11,00	17,00	16,00	13,00	4,00	15,00	6,00
0,00	1,00	7,00	7,00	0,00	8,00	15,00	12,00	18,00

0,00	1,00	19,00	12,00	9,00	8,00	10,00	13,00	15,00
0,00	1,00	11,00	9,00	5,00	9,00	1,00	20,00	15,00
0,00	1,00	15,00	10,00	7,00	20,00	12,00	11,00	20,00
0,00	1,00	14,00	15,00	16,00	11,00	15,00	11,00	16,00
1,00	1,00	16,00	15,00	12,00	4,00	4,00	11,00	15,00
0,00	1,00	16,00	7,00	10,00	4,00	3,00	20,00	15,00
0,00	1,00	15,00	15,00	10,00	13,00	9,00	7,00	17,00
0,00	4,00	19,00	18,00	20,00	19,00	0,00	16,00	9,00
0,00	2,00	13,00	13,00	13,00	20,00	8,00	19,00	19,00
1,00	3,00	2,00	0,00	12,00	4,00	0,00	19,00	12,00
0,00	5,00	6,00	6,00	0,00	3,00	7,00	16,00	7,00
1,00	2,00	14,00	15,00	16,00	11,00	6,00	17,00	4,00
#¡NULO!	2,00	14,00	5,00	15,00	10,00	1,00	18,00	5,00
1,00	2,00	12,00	10,00	8,00	20,00	1,00	17,00	4,00
0,00	2,00	12,00	10,00	13,00	7,00	1,00	17,00	9,00
1,00	2,00	11,00	18,00	7,00	19,00	1,00	17,00	7,00
0,00	1,00	9,00	18,00	12,00	8,00	8,00	16,00	4,00
0,00	1,00	12,00	17,00	14,00	13,00	4,00	7,00	2,00
1,00	1,00	20,00	8,00	4,00	0,00	1,00	14,00	17,00
0,00	1,00	15,00	18,00	20,00	3,00	9,00	15,00	12,00
0,00	1,00	20,00	19,00	12,00	16,00	1,00	20,00	7,00
0,00	3,00	12,00	10,00	12,00	14,00	8,00	17,00	10,00
0,00	2,00	20,00	19,00	15,00	16,00	7,00	16,00	0,00
0,00	1,00	14,00	15,00	15,00	13,00	12,00	6,00	1,00
0,00	2,00	6,00	14,00	12,00	4,00	10,00	16,00	1,00
1,00	3,00	20,00	20,00	19,00	18,00	9,00	4,00	3,00
0,00	4,00	11,00	17,00	15,00	11,00	9,00	13,00	4,00
0,00	#¡NULO!	19,00	13,00	14,00	13,00	9,00	19,00	15,00
0,00	3,00	8,00	5,00	8,00	7,00	5,00	17,00	12,00
0,00	3,00	13,00	7,00	9,00	9,00	1,00	19,00	9,00
0,00	5,00	14,00	9,00	15,00	9,00	3,00	17,00	2,00
0,00	2,00	10,00	6,00	12,00	9,00	2,00	10,00	16,00
0,00	2,00	16,00	15,00	18,00	14,00	4,00	15,00	11,00
0,00	1,00	15,00	7,00	11,00	9,00	9,00	8,00	14,00
0,00	1,00	7,00	12,00	12,00	6,00	14,00	10,00	17,00
1,00	1,00	20,00	18,00	20,00	19,00	9,00	20,00	4,00
1,00	2,00	4,00	15,00	8,00	14,00	2,00	20,00	7,00
0,00	2,00	10,00	2,00	4,00	8,00	3,00	17,00	17,00
0,00	2,00	2,00	4,00	9,00	5,00	4,00	20,00	13,00
0,00	2,00	14,00	15,00	15,00	14,00	7,00	20,00	6,00
0,00	3,00	16,00	5,00	13,00	8,00	1,00	20,00	10,00
0,00	2,00	16,00	11,00	16,00	0,00	2,00	19,00	5,00
0,00	2,00	5,00	7,00	9,00	6,00	11,00	17,00	12,00
0,00	#¡NULO!	19,00	13,00	16,00	12,00	4,00	19,00	7,00
0,00	3,00	20,00	10,00	11,00	9,00	0,00	19,00	6,00
0,00	3,00	13,00	5,00	12,00	4,00	0,00	18,00	13,00
0,00	2,00	16,00	15,00	13,00	4,00	2,00	10,00	6,00
0,00	3,00	15,00	10,00	15,00	13,00	2,00	17,00	6,00
0,00	2,00	9,00	5,00	8,00	14,00	7,00	6,00	7,00
0,00	3,00	11,00	17,00	15,00	11,00	9,00	13,00	4,00

0,00	3,00	17,00	11,00	14,00	15,00	3,00	16,00	4,00
0,00	2,00	20,00	0,00	15,00	16,00	4,00	0,00	0,00
0,00	2,00	15,00	14,00	18,00	10,00	4,00	8,00	3,00
1,00	4,00	0,00	6,00	0,00	4,00	12,00	20,00	19,00
0,00	4,00	18,00	18,00	12,00	3,00	7,00	16,00	4,00
0,00	3,00	20,00	11,00	17,00	14,00	0,00	20,00	10,00
0,00	3,00	13,00	15,00	15,00	6,00	2,00	5,00	2,00
0,00	4,00	7,00	9,00	10,00	6,00	3,00	19,00	8,00
1,00	3,00	18,00	8,00	17,00	13,00	0,00	16,00	13,00
0,00	2,00	15,00	12,00	6,00	13,00	4,00	19,00	8,00
0,00	1,00	20,00	7,00	20,00	8,00	4,00	20,00	4,00
0,00	1,00	12,00	7,00	16,00	6,00	7,00	10,00	13,00
0,00	1,00	16,00	8,00	20,00	14,00	9,00	7,00	10,00
0,00	3,00	15,00	6,00	11,00	10,00	5,00	15,00	11,00
0,00	5,00	17,00	12,00	10,00	11,00	6,00	14,00	10,00
0,00	4,00	16,00	15,00	12,00	17,00	5,00	18,00	11,00
0,00	5,00	10,00	7,00	2,00	4,00	0,00	17,00	20,00
0,00	4,00	20,00	10,00	15,00	7,00	0,00	18,00	8,00
0,00	1,00	20,00	16,00	12,00	4,00	4,00	12,00	19,00
0,00	1,00	10,00	11,00	4,00	3,00	8,00	15,00	18,00
0,00	1,00	14,00	8,00	14,00	8,00	6,00	10,00	14,00
0,00	1,00	11,00	3,00	6,00	12,00	2,00	4,00	12,00
0,00	3,00	16,00	18,00	16,00	19,00	0,00	17,00	5,00

CSI1_SC	CSI1_SPC	CSI2_PFE	CSI2_EFE	CSI2_PFD	CSI2_EFD	CSI3_E	CSI3_D
4,00	4,00	32,00	37,00	24,00	7,00	69,00	31,00
20,00	0,00	21,00	24,00	17,00	22,00	45,00	39,00
9,00	4,00	20,00	19,00	35,00	24,00	39,00	59,00
9,00	4,00	32,00	27,00	18,00	12,00	59,00	30,00
13,00	3,00	23,00	5,00	24,00	20,00	28,00	44,00
9,00	0,00	21,00	23,00	26,00	10,00	44,00	36,00
10,00	1,00	21,00	26,00	21,00	16,00	47,00	37,00
13,00	3,00	22,00	13,00	24,00	23,00	35,00	47,00
7,00	2,00	21,00	13,00	25,00	21,00	34,00	46,00
20,00	4,00	24,00	32,00	20,00	28,00	56,00	48,00
15,00	4,00	23,00	21,00	23,00	19,00	44,00	42,00
11,00	4,00	28,00	21,00	32,00	26,00	49,00	58,00
9,00	2,00	22,00	28,00	31,00	22,00	50,00	53,00
19,00	3,00	31,00	22,00	19,00	26,00	53,00	45,00
13,00	2,00	22,00	20,00	6,00	26,00	42,00	32,00
18,00	2,00	21,00	27,00	13,00	27,00	48,00	40,00
18,00	3,00	26,00	18,00	18,00	24,00	44,00	42,00
10,00	4,00	34,00	21,00	13,00	17,00	55,00	30,00
19,00	4,00	37,00	33,00	29,00	27,00	70,00	56,00
15,00	2,00	12,00	12,00	20,00	32,00	24,00	52,00
19,00	3,00	15,00	11,00	13,00	36,00	26,00	49,00
8,00	2,00	14,00	21,00	21,00	22,00	35,00	43,00
14,00	1,00	14,00	22,00	24,00	23,00	36,00	47,00
19,00	4,00	33,00	34,00	25,00	35,00	67,00	60,00
18,00	1,00	8,00	7,00	16,00	36,00	15,00	52,00
7,00	4,00	21,00	13,00	25,00	21,00	34,00	46,00
10,00	0,00	16,00	9,00	25,00	26,00	25,00	51,00
1,00	4,00	27,00	19,00	14,00	2,00	46,00	16,00
15,00	3,00	30,00	21,00	23,00	25,00	51,00	48,00
11,00	4,00	21,00	27,00	28,00	22,00	48,00	50,00
20,00	2,00	7,00	12,00	5,00	31,00	19,00	36,00
0,00	3,00	16,00	24,00	24,00	4,00	40,00	28,00
20,00	4,00	36,00	40,00	21,00	24,00	76,00	45,00
16,00	4,00	20,00	28,00	17,00	22,00	48,00	39,00
14,00	2,00	14,00	29,00	23,00	24,00	43,00	47,00
17,00	2,00	22,00	22,00	19,00	25,00	44,00	54,00
19,00	4,00	17,00	28,00	24,00	25,00	45,00	49,00
20,00	4,00	32,00	30,00	33,00	40,00	62,00	73,00
1,00	2,00	16,00	25,00	16,00	7,00	41,00	23,00
20,00	4,00	37,00	36,00	20,00	36,00	73,00	56,00
16,00	0,00	7,00	11,00	31,00	32,00	18,00	63,00
16,00	2,00	24,00	17,00	19,00	24,00	41,00	43,00
15,00	1,00	34,00	13,00	31,00	31,00	47,00	62,00
11,00	3,00	27,00	17,00	9,00	14,00	44,00	23,00
4,00	2,00	15,00	9,00	16,00	12,00	24,00	28,00
15,00	0,00	15,00	12,00	28,00	26,00	27,00	54,00
13,00	2,00	14,00	27,00	21,00	24,00	41,00	45,00
6,00	2,00	31,00	23,00	19,00	16,00	54,00	35,00
10,00	4,00	14,00	8,00	27,00	28,00	22,00	55,00

9,00	3,00	31,00	17,00	23,00	24,00	48,00	47,00
19,00	3,00	20,00	14,00	21,00	34,00	34,00	55,00
15,00	2,00	25,00	27,00	23,00	35,00	52,00	58,00
15,00	2,00	29,00	27,00	26,00	31,00	56,00	57,00
12,00	4,00	31,00	16,00	15,00	27,00	47,00	42,00
19,00	4,00	23,00	14,00	23,00	34,00	37,00	57,00
15,00	0,00	30,00	23,00	16,00	32,00	53,00	48,00
17,00	4,00	37,00	39,00	16,00	26,00	76,00	42,00
18,00	0,00	26,00	33,00	27,00	37,00	59,00	64,00
17,00	0,00	2,00	16,00	19,00	29,00	18,00	48,00
17,00	0,00	12,00	3,00	23,00	24,00	15,00	47,00
14,00	3,00	29,00	27,00	23,00	18,00	56,00	41,00
12,00	3,00	19,00	25,00	19,00	17,00	44,00	36,00
16,00	2,00	22,00	28,00	18,00	20,00	50,00	38,00
19,00	1,00	22,00	20,00	18,00	28,00	42,00	46,00
17,00	4,00	29,00	26,00	18,00	24,00	55,00	42,00
4,00	2,00	27,00	20,00	24,00	8,00	47,00	32,00
14,00	4,00	29,00	27,00	11,00	16,00	56,00	27,00
2,00	4,00	28,00	4,00	15,00	19,00	32,00	34,00
11,00	2,00	33,00	23,00	24,00	23,00	56,00	47,00
18,00	4,00	39,00	28,00	21,00	25,00	67,00	46,00
17,00	1,00	22,00	26,00	25,00	27,00	48,00	52,00
10,00	4,00	39,00	31,00	23,00	10,00	70,00	33,00
7,00	2,00	29,00	28,00	18,00	8,00	57,00	26,00
3,00	4,00	20,00	16,00	26,00	4,00	36,00	30,00
2,00	4,00	40,00	37,00	13,00	5,00	77,00	18,00
13,00	2,00	28,00	15,00	22,00	17,00	43,00	39,00
19,00	3,00	32,00	27,00	28,00	34,00	59,00	62,00
16,00	4,00	13,00	15,00	22,00	28,00	28,00	50,00
17,00	2,00	20,00	18,00	20,00	26,00	38,00	46,00
12,00	4,00	23,00	24,00	20,00	14,00	47,00	34,00
15,00	3,00	16,00	21,00	12,00	31,00	37,00	43,00
20,00	3,00	31,00	32,00	19,00	31,00	63,00	50,00
14,00	4,00	22,00	20,00	17,00	28,00	42,00	45,00
9,00	4,00	19,00	18,00	24,00	26,00	37,00	50,00
20,00	4,00	38,00	39,00	29,00	24,00	77,00	53,00
14,00	4,00	19,00	22,00	29,00	20,00	41,00	49,00
15,00	2,00	12,00	20,00	20,00	32,00	32,00	52,00
14,00	1,00	6,00	14,00	24,00	27,00	20,00	51,00
19,00	3,00	29,00	27,00	29,00	25,00	58,00	52,00
19,00	1,00	21,00	21,00	21,00	29,00	42,00	50,00
19,00	4,00	27,00	16,00	21,00	24,00	43,00	45,00
16,00	1,00	12,00	15,00	27,00	56,00	28,00	28,00
8,00	3,00	32,00	28,00	23,00	15,00	60,00	38,00
14,00	4,00	30,00	20,00	19,00	20,00	50,00	39,00
16,00	0,00	18,00	16,00	18,00	29,00	34,00	47,00
11,00	2,00	31,00	17,00	12,00	17,00	48,00	29,00
17,00	4,00	25,00	28,00	19,00	23,00	53,00	42,00
12,00	4,00	14,00	22,00	13,00	19,00	36,00	32,00
13,00	2,00	28,00	26,00	22,00	17,00	54,00	39,00

3,00	4,00	28,00	29,00	19,00	7,00	57,00	26,00
2,00	4,00	20,00	31,00	4,00	2,00	51,00	6,00
18,00	4,00	29,00	28,00	12,00	21,00	57,00	33,00
8,00	0,00	6,00	4,00	32,00	27,00	10,00	59,00
19,00	4,00	36,00	15,00	23,00	23,00	51,00	46,00
20,00	4,00	31,00	31,00	20,00	30,00	62,00	50,00
14,00	4,00	28,00	21,00	7,00	16,00	49,00	23,00
11,00	4,00	16,00	16,00	22,00	19,00	32,00	41,00
18,00	1,00	26,00	30,00	16,00	31,00	56,00	47,00
17,00	3,00	27,00	19,00	23,00	25,00	46,00	48,00
8,00	4,00	27,00	28,00	24,00	12,00	55,00	36,00
13,00	2,00	19,00	22,00	17,00	26,00	41,00	43,00
11,00	3,00	24,00	34,00	16,00	21,00	58,00	37,00
14,00	3,00	21,00	21,00	20,00	25,00	42,00	45,00
11,00	4,00	29,00	21,00	20,00	21,00	50,00	41,00
20,00	4,00	31,00	29,00	23,00	31,00	60,00	54,00
17,00	1,00	17,00	6,00	17,00	37,00	23,00	54,00
17,00	1,00	30,00	22,00	18,00	25,00	52,00	43,00
15,00	3,00	36,00	16,00	16,00	34,00	52,00	50,00
12,00	2,00	21,00	7,00	23,00	30,00	28,00	53,00
18,00	3,00	22,00	22,00	16,00	32,00	44,00	48,00
14,00	2,00	14,00	18,00	6,00	26,00	32,00	32,00
19,00	4,00	34,00	35,00	17,00	24,00	69,00	41,00
